# Examining bedtime procrastination, study engagement, and studyholism in undergraduate students, and their association with insomnia

**DOI:** 10.3389/fpsyg.2022.1111038

**Published:** 2023-01-17

**Authors:** Tahani K. Alshammari, Aleksandra M. Rogowska, Raghad F. Basharahil, Sumayyah F. Alomar, Sarah S. Alseraye, Lobna A. Al Juffali, Nouf M. Alrasheed, Musaad A. Alshammari

**Affiliations:** ^1^Department of Pharmacology and Toxicology, College of Pharmacy, King Saud University, Riyadh, Saudi Arabia; ^2^Institute of Psychology, University of Opole, Opole, Poland; ^3^College of Pharmacy, King Saud University, Riyadh, Saudi Arabia; ^4^Department of Clinical Pharmacy, King Fahad Medical City, Ministry of Health, Riyadh, Saudi Arabia; ^5^Department of Clinical Pharmacy, College of Pharmacy, King Saud University, Riyadh, Saudi Arabia

**Keywords:** insomnia, studyholism, bedtime procrastination, behavioral addiction, mental health, emerging adulthood, undergraduates education

## Abstract

**Introduction:**

Compulsive overstudying, known as studyholism, is an emerging behavioral addiction. In this study, we examine the prevalence of, and the relationships between, insomnia, study engagement, studyholism, bedtime procrastination among undergraduate students.

**Methods:**

The Studyholism (SI-10), Athens Insomnia (AIS), and bedtime procrastination scales were administered to a convenience sample of 495 university students.

**Results:**

Our findings indicate that the prevalence of insomnia was 75.31%, high studyholism was found in 15.31% of the sample, and increased study engagement was detected in 16.94%. Gender differences analysis revealed that females reported higher studyholism and bedtime procrastination than males. Fifth-year students had higher levels of studyholism than internship (*p* < 0.001), first-year (*p* < 0.01), and sixth-year students (*p* < 0.05). Insomnia was positively related to studyholism and bedtime procrastination. Furthermore, insomnia can be positively predicted by studyholism and bedtime procrastination. Participants with a medium level of studyholism were twice as likely to experience insomnia as those with a low level. Studyholics were six times more susceptible to insomnia than students with low studyholism levels. Compared to individuals with low bedtime procrastination levels, those with medium and high bedtime procrastination were twice as likely to report insomnia.

**Conclusion:**

Our study highlights the interplay between insomnia, studyholism, and bedtime procrastination. Further, the findings indicate the need to increase awareness of insomnia.

## 1. Introduction

Sleep is an essential physiological factor modulating body homeostasis (Buysse, 2014). However, approximately 45% of adolescents experience insufficient sleep (Ming et al., 2011). Furthermore, a significant number of undergraduate students report poor sleep quality (Taylor et al., 2013; Pensuksan et al., 2016; Seun-Fadipe and Mosaku, 2017; Shrestha et al., 2021). Insomnia is linked to psychological stress, reduced academic performance, elevated risks of depression and anxiety (Paulina Ojeda-Paredesa and Rubio-Zapataa, 2019; Alshammari et al., 2022b), and risk-taking behavior (Moore and Meltzer, 2008). Insomnia has been recognized as a significant health issue negatively affecting cognitive performance, mood (Alhola and Polo-Kantola, 2007), immune function (Irwin, 2015), cardiovascular risk (Kwok et al., 2018), weight, and metabolism (Ming et al., 2011; Papatriantafyllou et al., 2022). Insomnia is a health burden in undergraduate students that requires comprehensive assessment and an understanding of factors contributing to its pathology and increased prevalence.

Bedtime procrastination is defined as the psychological condition of purposely delaying bedtime with no external circumstances (Chow, 2011). It is a complex experience composed of both affective and cognitive aspects, reflecting poor self-control (Kroese et al., 2014b). The procrastination construct is built on multiple domains, including reduced conscientiousness and self-regulatory capacities, and impulsivity (Kroese et al., 2014a). Previous studies have linked bedtime procrastination to fatigue and poor sleep quality (Kroese et al., 2014a), depression, smartphone addiction (Geng et al., 2021), anxiety (Türkarslan et al., 2020), and insomnia (Hammoudi et al., 2021). Hairston and Shpitalni (2016) found that bedtime procrastination is associated with ruminative cognition and sleep difficulties, while Sirois et al. (2015) reported that procrastination is associated with multiple dimensions of sleep quality. Bedtime procrastination has been linked to reduced sleep duration and lower sleep quality (Li et al., 2020). In terms of mood, it has been associated with anxiety (Rubin, 2020), depression (Geng et al., 2021), and social jetlag (Li et al., 2020).

In a Polish study, it was reported that bedtime procrastination is higher in students than in non-students (Herzog-Krzywoszanska and Krzywoszanski, 2019). This highlights the unmet need to examine bedtime procrastination in students’ lives. Several studies have linked bedtime procrastination to behavioral addiction, such as internet addiction (Reinecke et al., 2018; You et al., 2020), social media addiction (Sai et al., 2020; Exelmans and Van den Bulck, 2021), and smartphone addiction (Zhang and Wu, 2020; Geng et al., 2021; Chen et al., 2022; Mao et al., 2022). However, to the best of our knowledge, no current study has examined the link between bedtime procrastination and studyholism, which is more fundamental in students’ lives. Only one study has examined procrastination in the Saudi population (Dardara and Al-Makhalid, 2022), but the research was conducted on students from a single institute. Thus, the role and prevalence of bedtime procrastination are far from being determined in the Saudi community.

Studyholism is defined as an emerging clinical condition similar to an obsessive–compulsive related disorder which might be associated with either high or low levels of study engagement (SE). Conceptually, it is built on a three-item construct: (1) addiction-like symptoms (externalizing); (2) obsessive–compulsive related symptoms (internalizing); and (3) study engagement (Loscalzo and Giannini, 2020). It is essential to understand the mechanisms and risk factors related to this study-related compulsive condition. Studyholism might emerge due to exam-related anxiety, a preoccupation with receiving an unsatisfactory grade. However, pathological compulsion behavior could develop in students, leading to a clinically significant condition.

Notably, while other types of behavioral addiction, such as internet gaming disorder, are viewed as negative conditions (Hou et al., 2019); both workaholism and studyholism are considered positive traits (Atroszko et al., 2015). Recently, however, both workaholism and studyholism have been recognized as problematic. Previous reviews have linked workaholism with social relationship problems, life dissatisfaction, lower levels of well-being, psychological distress, and depression (Quinones and Griffiths, 2015; Loscalzo and Giannini, 2017). Nevertheless, current evidence is sparse, especially in the context of studyholism (Loscalzo and Giannini, 2021). Further insights are needed, including an analysis of correlates linked to studyholism (Loscalzo and Giannini, 2018). Based on the fact that bedtime procrastination, insomnia, and addictive behavior are connected (You et al., 2020; Zhang and Wu, 2020; Hammoudi et al., 2021), this study investigated the prevalence and the relationship between study engagement, studyholism, bedtime procrastination, and insomnia in undergraduate students from Saudi institutes. The central goal was to examine the prevalence and the association of study engagement and studyholism with behavioral factors such as insomnia and bedtime procrastination. This is the first report to examine the association among bedtime procrastination and studyholism in a student sample. Our analysis considers the effect of multiple variables, including gender, study year, and specialty track. The research instruments employed were the Studyholism (SI-10), Athens Insomnia (AIS), and bedtime procrastination scales.

## 2. Materials and methods

### 2.1. Participants

A total of 495 students aged between 18 and 33 years (*M* = 20.89, SD = 2.01) participated in the study (Table 1). The sample comprised 187 men and 303 women, most with families consisting of 7 members. Participants represented universities from all geographical regions of Saudi Arabia, all study years, and specialties (Table 1).

**Table 1:** Demographic characteristics of the sample (*N* = 495).

Variable	Categories	*n*	%
Gender	Female	303	61.84
	Male	187	38.16
Marital Status	Married	14	2.86
	Single	476	97.14
Geographical region	The Central Region (Riyadh, Qasim)	333	67.96
	The Eastern Region (Damam, Khafji, Alhasa)	47	9.59
	The Northern Region (Tabuk, Jouf, Hail)	25	5.10
	The Southern Region (Asir, Najran, Jizan)	36	7.35
	The Western Region (Mecca, Medina, Jeddah)	49	10.00
Number of family members	0–4	54	11.02
	5	58	11.84
	6	86	17.55
	7	116	23.67
	8	67	13.67
	9	51	10.41
	10 or more	58	11.84
Study year	Internship	80	16.33
	First	87	17.76
	Second	85	17.35
	Third	78	15.92
	Fourth	96	19.59
	Fifth	45	9.18
	Sixth	19	3.88
Specialty track	Applied and community service	16	3.27
	Business Administration college	128	26.12
	Health track	226	46.12
	Humanities track	20	4.08
	Nursing college	22	4.49
	Science track	78	15.92

### 2.2. Study design and procedure

A cross-sectional study was conducted with male and female students recruited *via* convenience sampling at various universities across the Kingdom of Saudi Arabia from the 16th of April to the 3rd of June, 2022. Google forms in English were used to create an online questionnaire distributed to participants through Twitter, WhatsApp, Telegram, and so on. At the start of the survey, participants were provided with a consent participation message, a description of the project aims, and a reassurance that their responses were confidential and completely voluntary. Ethical approval was granted from the Institutional Review Board at King Saud University in Riyadh, Saudi Arabia (Ref. No. 22/0380/IRB).

### 2.3. Measurements

#### 2.3.1. Insomnia

The Athens Insomnia Scale (AIS) is a tool used to evaluate insomnia. It is a self-assessment psychometric instrument designed for quantifying sleep difficulty based on the ICD-10 criteria. It consist of eight items covering multiple domains of sleep characteristics, including sleep induction, awakenings during the night, final awakening, total sleep duration, and sleep quality. Additionally, it examines well-being, functioning capacity, and sleepiness during the day (Soldatos et al., 2000). A cut-off score of 6 or more was used for recognizing insomnia (Soldatos et al., 2003). The reliability of the AIS in the current study was Cronbach’s *α* = 0.76.

#### 2.3.2. Studyholism

The SI-10 is a valid instrument with psychometric properties related to study habits. It starts with questions addressing study habits such as hours of study per day and at the weekend, followed by ten items that evaluate Studyholism and engagement in studying behavior. Participants’ responses were recorded on a 5-point Likert scale ranging from 1 (*Strongly disagree*) to 5 (*Strongly agree*). A sample item for the Studyholism (SH) scale is “I cannot relax because of worries about studying,” and a sample item for the Study Engagement (SE) scale is “I study very hard to get the best grades.” In this work, we utilized the English 10-item version (Loscalzo and Giannini, 2020). The scores are categorized as low (4–9 for SH, and 4–10 for SE), medium (10–18 for SH and 11–18 for SE), and low (19–20 for SH and SE) (Loscalzo and Giannini, 2020). The internal consistency (Cronbach’s α) in the current study of SH and SE scales was 0.87 and 0.89, respectively.

#### 2.3.3. Bedtime procrastination

Bedtime procrastination was evaluated using the Bedtime Procrastination Scale developed by Kroese and colleagues. This is a nine-item instrument (e.g., “I go to bed early if I have to get up early in the morning”), and items were answered on a five-point Likert-like scale ranging from 1 (never) to 5 (always). Total scores ranged between 9 and 45. Higher scores represent a higher prevalence of bedtime procrastination (Magalhães et al., 2020). The reliability coefficient in the present study was Cronbach’s *α* = 0.64.

#### 2.3.4. Demographics

The demographic survey included questions about age (years), gender (man, woman), marital status (married, single), geographical regions of Saudi Arabia (Central, Eastern, Northern, Southern, and Western), number of family members (0–4, 5, 6, 7, 8, 8, 10 or more), study year (internship, first, second, third, fourth, fifth, sixth), and specialty track (applied and community service, business administration college, health track, humanities track, nursing college, and science track).

### 2.4. Statistical analysis

First, descriptive statistics were generated, namely range of scores (minimum and maximum), mean (*M*), standard deviation (*SD*), median (*Mdn*), skewness, and kurtosis. Because the sample size was quite large (*N* = 490) and skewness and kurtosis ranged between-0.50 and 0.50, parametric tests were performed to asses gender differences (Student’s t-test), differences between study years (one-way analysis of variance, ANOVA), and associations between insomnia, studyholism, study engagement, and bedtime procrastination (Pearson’s correlations). The predictors of insomnia (cut-off 6) were examined using binomial logistic regression. All statistical tests were performed using the JASP software, ver. 0.16.1 (University of Amsterdam, 2013–2022).

## 3. Results

### 3.1. Prevalence of insomnia, studyholism, and bedtime procrastination

Insomnia was prevalent in 75.31% (*n* = 369) of students, with a cut-off score of 6 or more (suitable for the general population). High studyholism was found in 15.31% (*n* = 75) participants, while high study engagement was found in 16.94% (*n* = 83) (see Table 2 for details). Taking into consideration low and high scores in SH and SE, there were 81 (16.53%) detached students, one disengaged studyholic (0.20%), 5 engaged students (1.02%), and 29 engaged studyholics (5.92%). Most students (374, 76.33%) were not included in any of the above, as they presented medium scores in one or both studyholism and SE scales. As indicated in Tables 2, 84 participants (17.14%) had high bedtime procrastination scores.

**Table 2:** Frequencies of low, medium, and high scores in studyholism, study engagement, and bedtime procrastination.

Variable	Descriptives (*N* = 495)		Low		Medium		High	
	*M*	*SD*	*n*	%	*n*	%	*n*	%
Studyholism (SH)	13.14	4.65	124	25.31	291	59.39	75	15.31
Study engagement (SE)	13.41	4.64	133	27.14	274	55.92	83	16.94
Bedtime procrastination (BPS)	29.45	6.03	64	13.06	342	69.80	84	17.14

Studyholism (SH), Study engagement (SE), Bedtime procrastination (BPS).

### 3.2. Gender differences

The parametric properties of scales were initially examined using range of scores, median (*Mdn*.), mean (*M*), standard deviation (*SD*), skewness, and kurtosis. The descriptive statistics (Table 3) revealed good psychometric characteristics of all variables given the large sample size (*N* = 490). Therefore, Student’s *t-features* of the dataset, and one-way ANOVA were performed to assess intergroup differences.

**Table 3:** Descriptive statistics (*N* = 495).

Variable	Range	*Mdn.*	*M*	*SD*	Skewness	Kurtosis
Studyholism (SH)	4–20	13	13.14	4.65	***−***0.26	***−***0.89
Study engagement (SE)	4–20	14	13.41	4.64	***−***0.35	***−***0.85
Insomnia (AIS)	0–24	9	8.72	4.46	0.27	***−***0.18
Bedtime procrastination (BPS)	10–45	28	29.45	6.03	0.18	0.14

As Table 4 reveals, women reported higher scores than men in studyholism and bedtime procrastination. However, the effect size is weak for both comparisons. No significant gender differences were found for study engagement and insomnia.

**Table 4:** Student’s *t*-test for gender differences in studyholism, study engagement, insomnia, and bedtime procrastination (*N* = 495).

Variables	Women (*n* = 303)		Men (*n* = 187)		*t*(488)	*p*	*d*
	*M*	*SD*	*M*	*SD*			
Studyholism (SH)	13.53	4.76	12.52	4.42	2.34	0.020	0.22
Study engagement (SE)	13.58	4.74	13.14	4.48	1.03	0.303	0.10
Insomnia (AIS)	8.70	4.54	8.77	4.35	***−***0.18	0.859	***−***0.02
Bedtime procrastination (BPS)	29.92	6.24	28.68	5.60	2.23	0.026	0.21

### 3.3. Study year differences in studyholism

The differences between groups of students from various years of study for studyholism and study engagement were examined separately using one-way ANOVA (Table 5). We found significant differences in studyholism, but not in study engagement. Post-hoc Bonferroni comparisons indicated that fifth year students scored significantly higher in studyholism than internship (*p* < 0.001), first year (*p* < 0.01), and sixth year students (*p* < 0.05). No other differences were significant.

**Table 5:** One-way ANOVA for differences in studyholism and study engagement between students representing various study years (*N* = 495).

Study year	*n*	Studyholism		Study engagement	
		*M*	*SD*	*M*	*SD*
Internship	80	11.75	5.01	13.21	5.24
First	87	12.17	4.39	13.43	4.85
Second	85	13.59	4.72	13.26	4.50
Third	78	13.74	4.66	14.31	4.58
Fourth	96	13.52	4.11	12.79	4.36
Fifth	45	15.44	4.38	14.20	3.49
Sixth	19	11.63	4.61	12.53	5.42
*F*		4.64		1.15	
*p*		<0.001		0.335	
*η* ^2^		0.06		0.01	

### 3.4. Associations between variables

**Figure 1:**
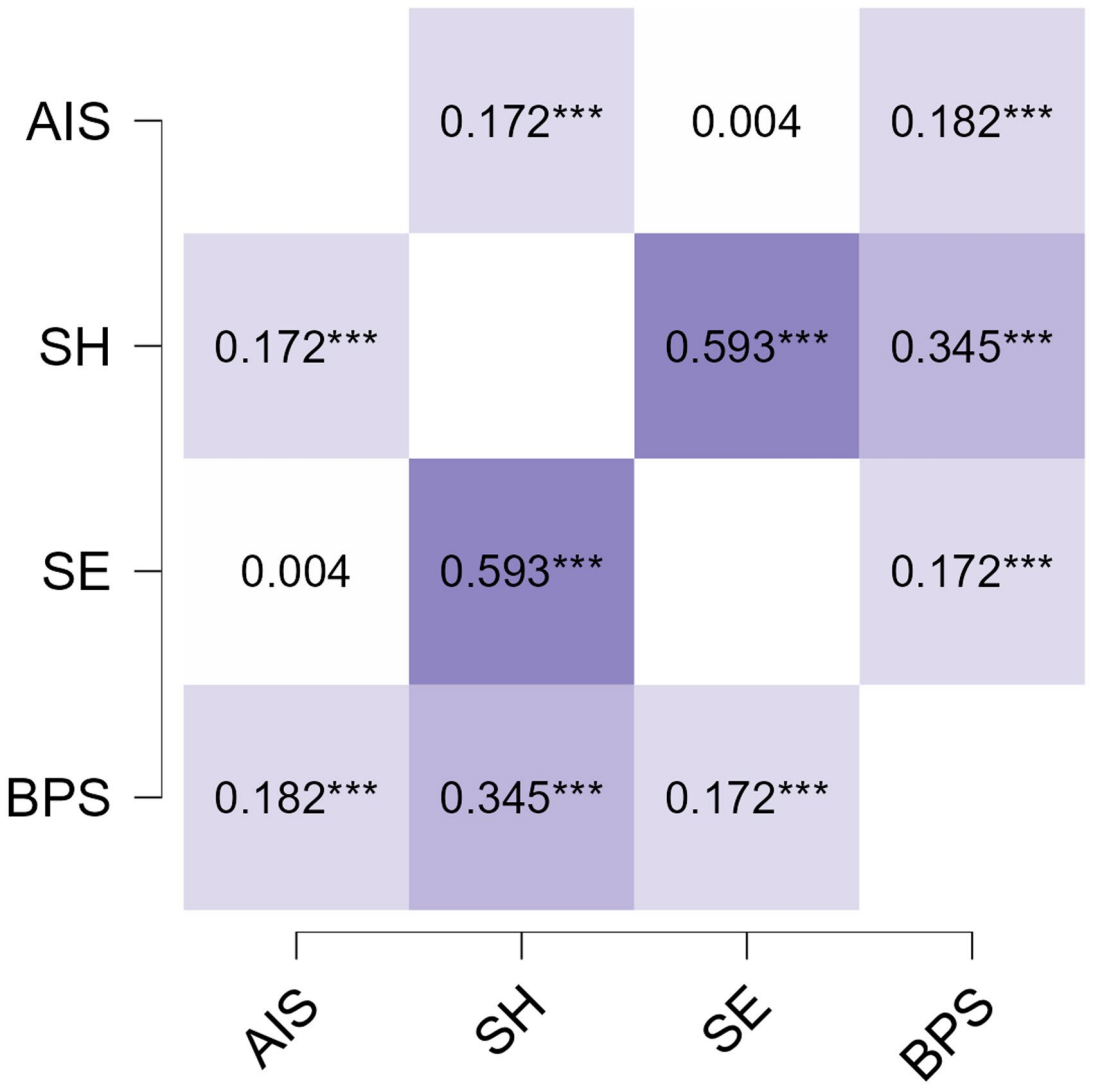
Pearson’s correlations between insomnia (AIS), studyholism (SH), study engagement (SE), and bedtime procrastination (BPS). ***p < 0.001.

The associations between insomnia, studyholism, study engagement, and bedtime procrastination are presented in Figure 1. Insomnnia was positively but weakly related to studyholism and bedtime procrastination, but was not related to study engagement. A moderate association was found between studyholism and study engagement. Bedtime procrastination was weakly related to both studyholism and SE. All associations were statistically significant (*p* < 0.001).

A binomial logistic regression was performed to examine whether the demographic variables, studyholism, study engagement, and bedtime procrastination were predictors of insomnia. Firstly, the associations between insomnia and each independent variable were calculated separately using crude odds ratios (Table 3). Among all independent variables, only studyholism and bedtime procrastination were found to be significant predictors of insomnia (Table 6). None of the demographic variables were significant predictors of insomnia.

**Table 6:** Binomial logistic regression for insomnia (crude odds ratios).

Predictor	*B*	LL	UL	*SE*	*Z*	*p*	OR	LL	UL
Gender: Women (ref. Men)	−0.10	−0.53	0.32	0.22	−0.47	0.639	0.90	0.59	1.38
Marital status: Married (ref. Single)	0.55	−0.57	1.66	0.57	0.96	0.337	1.72	0.57	5.25
Number of family members (ref. 0–4)									
5	0.88	−0.08	1.83	0.49	1.80	0.072	2.40	0.93	6.25
6	0.05	−0.71	0.82	0.39	0.13	0.894	1.05	0.49	2.26
7	0.44	−0.31	1.19	0.38	1.15	0.249	1.56	0.73	3.29
8	0.05	−0.76	0.85	0.41	0.11	0.911	1.05	0.47	2.34
9	0.46	−0.46	1.37	0.47	0.98	0.328	1.58	0.63	3.93
10 or more	−0.61	−1.40	0.19	0.40	−1.50	0.133	0.55	0.25	1.20
University region (ref. Central)									
Eastern	−0.22	−0.91	0.46	0.35	−0.64	0.525	0.80	0.40	1.59
Northern	−0.78	−1.62	0.06	0.43	−1.82	0.069	0.46	0.20	1.06
Southern	0.43	−0.49	1.34	0.47	0.91	0.361	1.53	0.61	3.81
Western	−0.27	−0.94	0.40	0.34	−0.79	0.432	0.77	0.39	1.49
Study year (ref. Internship)									
First	0.38	−0.35	1.11	0.37	1.03	0.303	1.47	0.71	3.03
Second	−0.32	−1.00	0.35	0.34	−0.94	0.348	0.73	0.37	1.42
Third	0.10	−0.62	0.82	0.37	0.27	0.785	1.11	0.54	2.27
Fourth	0.50	−0.22	1.23	0.37	1.36	0.172	1.65	0.80	3.41
Fifth	−0.24	−1.04	0.57	0.41	−0.58	0.562	0.79	0.35	1.76
Sixth	0.00	−1.14	1.13	0.58	−0.01	0.995	1.00	0.32	3.10
Specialty track (ref. Health track)									
Applied and community service	−0.28	−1.60	1.04	0.67	−0.42	0.675	0.75	0.20	2.82
Business Administration college	−0.43	−1.72	0.86	0.66	−0.65	0.518	0.65	0.18	2.37
Humanities track	−0.37	−1.98	1.25	0.82	−0.45	0.655	0.69	0.14	3.47
Nursing college	−0.49	−2.05	1.08	0.80	−0.61	0.544	0.62	0.13	2.95
Science track	−0.26	−1.62	1.10	0.70	−0.38	0.706	0.77	0.20	3.00
Studyholism	0.07	0.02	0.11	0.02	2.93	0.003	1.07	1.02	1.12
Study engagement	−0.02	−0.07	0.02	0.02	−0.95	0.344	0.98	0.94	1.02
Bedtime procrastination	0.07	0.03	0.11	0.02	3.65	< 0.001	1.07	1.03	1.11

Secondly, the entire regression model was performed for all independent variables included simultaneously in the regression analysis, using the adjusted odds ratio (Table 7). The assumptions for logistic regression were met as the variance inflation factor (VIF) ranged between 1.04 and 1.15, while tolerance ranged between 8.87 and 0.96. As indicated in Table 7, no demographic variables were significant predictors of insomnia. Insomnia was, however, positively predicted by studyholism and bedtime procrastination. Participants with a medium level of studyholism were twice as likely to experience insomnia than those with a low level. Studyholics (those with high SH scores) were six times more susceptible to insomnia than students with low studyholism levels. Compared to individuals with low bedtime procrastination levels, those with medium and high bedtime procrastination were twice as likely to report insomnia. The overall regression model was significant, but explained only 10% of insomnia variance (McFadden’s *R*^2^ = 0.10, *Χ*^2^(29) = 56.91, *p* < 0.001).

**Table 7:** Binomial logistic regression for insomnia (adjusted odds ratios).

Predictor	*B*	LL	UL	*SE*	*Z*	*p*	AOR	LL	UL
Intercept	−0.43	−1.95	1.10	0.78	−0.55	0.584	0.65	0.14	2.99
Gender: Women (ref. Men)	−0.15	−0.63	0.33	0.25	−0.61	0.540	0.86	0.53	1.39
Marital status: Married (ref. Single)	−0.46	−1.84	0.91	0.70	−0.66	0.510	0.63	0.16	2.49
Number of family members (ref. 0–4)									
5	0.53	−0.52	1.58	0.54	0.99	0.321	1.70	0.60	4.87
6	−0.04	−0.90	0.81	0.44	−0.10	0.922	0.96	0.41	2.25
7	0.21	−0.64	1.06	0.44	0.49	0.626	1.24	0.53	2.90
8	−0.18	−1.08	0.72	0.46	−0.39	0.694	0.84	0.34	2.05
9	0.18	−0.82	1.18	0.51	0.36	0.719	1.20	0.44	3.27
10 or more	−0.76	−1.67	0.15	0.46	−1.65	0.100	0.47	0.19	1.16
University region (ref. Central)									
Eastern	−0.06	−0.85	0.74	0.41	−0.14	0.886	0.94	0.43	2.09
Northern	−0.56	−1.51	0.40	0.49	−1.14	0.254	0.57	0.22	1.49
Southern	0.95	−0.08	1.97	0.52	1.81	0.070	2.57	0.92	7.17
Western	−0.18	−0.93	0.57	0.38	−0.48	0.634	0.84	0.40	1.76
Study year (ref. Internship)									
First	0.20	−0.61	1.01	0.41	0.49	0.623	1.23	0.55	2.75
Second	−0.69	−1.46	0.07	0.39	−1.77	0.077	0.50	0.23	1.08
Third	−0.20	−1.00	0.61	0.41	−0.48	0.631	0.82	0.37	1.83
Fourth	0.20	−0.59	0.98	0.40	0.49	0.622	1.22	0.56	2.66
Fifth	−0.66	−1.56	0.24	0.46	−1.45	0.149	0.52	0.21	1.27
Sixth	−0.24	−1.54	1.06	0.66	−0.36	0.716	0.79	0.22	2.88
Specialty track (ref. Health track)									
Applied and community service	0.31	−1.08	1.69	0.71	0.44	0.663	1.36	0.34	5.41
Business Administration college	0.14	−0.46	0.74	0.31	0.46	0.645	1.15	0.63	2.10
Humanities track	0.01	−1.20	1.23	0.62	0.02	0.986	1.01	0.30	3.41
Nursing college	−0.01	−1.10	1.08	0.56	−0.02	0.983	0.99	0.33	2.93
Science track	0.14	−0.53	0.80	0.34	0.40	0.688	1.15	0.59	2.23
Studyholism	0.12	0.06	0.19	0.03	3.66	< 0.001	1.13	1.06	1.21
Study engagement	−0.10	−0.16	−0.03	0.03	−3.05	0.002	0.91	0.86	0.97
Bedtime procrastination	0.05	0.01	0.09	0.02	2.53	0.011	1.05	1.01	1.10

## 4. Discussion

In this study we examined the correlations between insomnia, studyholism, and bedtime procrastination. Because insomnia is a clinical condition and a serious risk factor for mental health disorders, we focused on elucidating factors associated with insomnia. Our findings indicated that the prevalence of insomnia was 75.31%, high studyholism was found in 15.31% of the sample, and high study engagement in 16.94%. Insomnia was positively related to studyholism and bedtime procrastination. Our data indicated that women scored higher than men in studyholism and bedtime procrastination. The one-way ANOVA revealed that fifth year students scored significantly higher in studyholism. A moderate association was found between studyholism and study engagement. Bedtime procrastination was weakly associated with both studyholism and study engagement. These associations were found to be significant using Pearson’s correlation. Furthermore, insomnia was positively predicted by studyholism and bedtime procrastination. Participants with a medium level of studyholism were twice as likely to experience insomnia than those with a low level. Studyholics (those with high SH scores) were six times more susceptible to insomnia than students with low studyholism levels. Compared to individuals with low bedtime procrastination levels, those with medium and high bedtime procrastination were twice as likely to report insomnia.

We identified a high prevalence of insomnia among participants. In support of our findings, previous reports utilizing the Pittsburgh Sleep Quality Index (PSQI) have reported a significant number of students exhibiting insomnia (Lund et al., 2010; Schlarb et al., 2017). Schlarb et al. reported that more than 70% of their participants reported some domains of insomnia, and 50% reported insomnia symptoms that meet the fifth edition of the Diagnostic and Statistical Manual of Mental Disorders criteria. A recent report by AlHadi and Alhuwaydi (2022) examined insomnia in students from Saudi universities during the COVID-19 pandemic lockdown. Their findings indicated that insomnia was present in approximately 41% of participants. Several differences exist, though. First, we utilized different scales, while in their study, they used the Insomnia Severity Scale (ISI). More importantly, they carried out their study during COVID-19. In support of this, Wright et al. (2020) revealed that sleep patterns differed pre-and during the pandemic suggesting that human behavior, including sleeping patterns and quality, changes during pandemics. A systematic review and meta-analysis revealed that the global burden of sleep disturbances during the pandemic was approximately 40%. Nearly four in every ten people presented with some domain of sleep problem. In addition, the sleep disturbances were different in situations with or without lockdown (Jahrami et al., 2022). This was supported by another systematic and meta-analysis review, which found that the pandemic affected both sleep quality and insomnia prevalence. Notably, this review highlighted the female gender as a strong predictor for insomnia (Scarpelli et al., 2022). In our report, the majority of participants were female and single, which rendered gender a contributing factor to the high prevalence of insomnia. Indeed, previous studies have linked a higher prevalence of insomnia with the female gender and being single (Morin et al., 2011; Jung and Lee, 2016; Duran and Kaynak, 2022; Tsou, 2022). Additionally, poor sleep quality is associated with gender and emotional regulation (Alfonsi et al., 2022). Lifestyle can also impact sleep patterns and habits (Shochat, 2012). For instance, Lund et al. (2010) reported that insomnia prevailed in more than 60% of their study population, which was attributed to the use of electronics and frequency of exercise.

Our findings indicated that bedtime procrastination was higher in females than males. This contrasted with a previous report conducted by Dardara and Al-Makhalid (2022) on the Saudi population which reported that males exhibited procrastination more than females. However, this study investigated procrastination in a single Saudi institute, Umm Al-Qura University. Also, they examined procrastination behavior rather than bedtime procrastination and utilized a different tool, the Irrational Procrastination Scale (IPS). These factors might contribute to the discrepancies between our study and the previous report. On the other hand, another study conducted in a Polish sample reported that females scored higher in bedtime procrastination (Herzog-Krzywoszanska and Krzywoszanski, 2019).

Pearson’s correlations indicated that insomnia was positively but weakly related to bedtime procrastination, and that individuals with medium and high bedtime procrastination were twice as likely to report insomnia. Bedtime procrastination was a predictor of both the prevalence and the severity of poor sleep quality. Research by Ma et al. (2022) on a sample of Chinese undergraduate students revealed that participants with high bedtime procrastination were twice as susceptible to insomnia.

Study engagement reflects the positive aspect of studyholism. Conversely, studyholism is more of a negative construct, reflecting problematic overstudying, a feature of OCD (Loscalzo and Giannini, 2021). Our findings indicate that high studyholism was found in 15.31% of participants and high study engagement in 16.94%. Furthermore, a higher level of studyholism was reported in females, which could be attributed to the fact that females exhibit higher levels of perfectionism in university performance (Haase et al., 2013). Another contributing factor is that emotional dysregulation has been reported to be relevant to other types of addiction behavior, such as binge-watching behavior (Alfonsi et al., 2022) and problematic internet use (Usubini et al., 2022).

Using the anxiety subscale of DASS-42, Marina and Mamić (2020) linked studyholism to anxiety. Another study of an adolescent sample indicated that studyholism is positively predicted by social anxiety, furthering the notion that studyholism is an OCD-related condition (Loscalzo and Giannini, 2022). Notably, previous research using the perceived academic control scale with Croatian college students indicated a lack of correlation between studyholism and academic performance (Marina and Mamić, 2020).

Accumulated evidence has described and analyzed different forms of addiction behavior in college students, including gaming addiction (Yao et al., 2015; Liu et al., 2016), social media addiction (Sai et al., 2020; Exelmans and Van den Bulck, 2021), online social networking addiction (Tang and Koh, 2017; Azizi et al., 2019), and smartphone addiction (Zhang and Wu, 2020; Geng et al., 2021; Chen et al., 2022; Mao et al., 2022). These types of addictions have been examined to determine whether they are comorbid with other conditions (Tang and Koh, 2017), affect higher cognitive tasks such as decision-making (Yao et al., 2015), or are linked to the level of academic adjustment (Sai et al., 2020). Additionally, their interplay with affective disorders, including depression and anxiety, has been examined (Haand and Shuwang, 2020; Keles et al., 2020; Nguyen et al., 2020). However, research on the concept of studyholism and risk factors that predict and/or link it to other behavioral conditions is underrepresented.

Pearson’s correlations between insomnia and other variables indicated that insomnia was positively related to studyholism, but was not related to study engagement. Furthermore, the binomial logistic regression revealed that insomnia could be positively predicted by studyholism and bedtime procrastination. Participants with a medium level of studyholism were twice as likely to experience insomnia than those with a low level. Studyholics (those with high SH scores) were six times more susceptible to insomnia than those with low studyholism levels. Further, the correlation analysis between insomnia and studyholism can be anticipated by two notions. The first is that stress regarding school-related problems and intolerance of uncertainty affect sleep quality (Lin et al., 2017). Second, overstudying shares multiple features with workaholism, such as perfectionism and conscientiousness (Loscalzo and Giannini, 2018). Workaholism has been found to be related to an elevated risk of sleep problems in nurses (Kubota et al., 2010), including insufficient sleep, daytime sleepiness, and impaired awakening. In a Japanese sample of a 7-month follow-up study, it was found that high-workaholic employees exhibited longer sleep latency than low-and moderate-workaholic employees (Kubota et al., 2014). Moreover, in a mediation model, it was reported that smartphone-intensive use mediates the association between poor sleep quality and workaholism (Spagnoli et al., 2019). This indicates complex mechanisms exist between workaholism and sleep disorders. In line with this, another report indicated that workaholism significantly mediates multiple health outcomes, including insomnia, social dysfunction, and anxiety (Andreassen et al., 2018). Overall, our regression analysis was significant, and the variables accounted for 10% of insomnia variance, indicating the complex nature of insomnia and sleep disorders.

Our one-way ANOVA revealed that fifth year students scored significantly higher in studyholism. Conceptually, studyholism is identified by negative emotions and overall stress (Loscalzo, 2021), and we anticipated this effect could be driven by pre-graduation stress. Students could experience more pressure to improve their academic performance before the sixth year or internship, where improving their GPA is quite difficult. We previously reported that the study year is a stress-related risk factor. A study examining stress toward virtual learning among students from colleges of health sciences found that the risk of stress is twice as high in first-third year students than internship students (Alshammari et al., 2022a). Another study investigated the prevalence and risk factors of insomnia, stress, anxiety, and depression in a sample of Egyptian medical students (Barakat et al., 2016). The authors found that approximately 40% of fifth-year medical students face difficulties coping with academic demands. In line with this, previous reports identified study year as a factor contributing to depression (Chen et al., 2018; Wagner et al., 2022), stress, anxiety (Barakat et al., 2016), procrastination (Ibrahim et al., 2021), and insomnia (Roka et al., 2020).

### 4.1. Limitations of the study

Although a significant relationship was found between insomnia, bedtime procrastination, and studyholism, a number of limitations prevent generalization of these results. First, we used a convenience sample of university students for the online survey, hence the results may not be representative of the entire population of university students. Additionally, the lower alpha of the measures limits the generalization of the study results. Also, the sample was dominated by women and students representing the Central Region of Saudi Arabia who were studying a health track and business and administration. Future studies should include a more gender balanced and representative university sample.

In addition, the overall regression model was significant but explained only 10% of insomnia variance. This means that other variables, not included in the regression model, may be more important for insomnia than BP, SH, and demographics. For example, we did not control for well-being dimensions such as the severity of generalized anxiety disorder, social anxiety, depression, PTSD, or OCD. Therefore, future studies should consider the inclusion of physical and mental health dimensions as variables. Also, the reliability of the Bedtime Procrastination Scale was poor, so cultural adaptation of the measure is required. Furthermore, studyholism is a controversial behavioral addiction. Some limitations exist in our study design, such as recall bias, desirability bias, and common method bias. Another limitation of our methods is that we cannot estimate the response rate. Finally, we used a cross-sectional study design so the causal effects should be treated with caution. Longitudinal studies are required to confirmed the predictive value of SH and BP for insomnia.

## 5. Conclusion

This report highlighted the significant prevalence of insomnia. Further, it identified an association between insomnia, bedtime procrastination, and studyholism in undergraduate students. The findings also highlighted gender and study-year as risk factors for studyholism. Given these results, efforts should be directed toward establishing institutional insomnia awareness programs and educational workshops aimed at maintaining good sleep quality, especially in females and students who face difficulties coping with stress.

## Data availability statement

The raw data supporting the conclusions of this article will be made available by the authors, without undue reservation.

## Ethics statement

The studies involving human participants were reviewed and approved by Institutional Review Board at King Saud University in Riyadh, Saudi Arabia (Ref. No. 22/0380/IRB). The patients/ participants provided their consent to participate in this study.

## Author contributions

TA and AR: conceptualization, methodology, and writing—review and editing. AR: formal analysis and data curation. RB, SFA, and SSA: investigation. TA: resources and funding acquisition. TA, RB, SFA, LA, and AR: writing—original draft preparation. MA and NA: supervision. All authors have read and agreed to the published version of the manuscript.

## Funding

The authors extend their appreciation to the Deputyship for Research & Innovation, Ministry of Education in Saudi Arabia for funding this research work through the project no. (IFKSURG-1095).

## Conflict of interest

The authors declare that the research was conducted in the absence of any commercial or financial relationships that could be construed as a potential conflict of interest.

## Publisher’s note

All claims expressed in this article are solely those of the authors and do not necessarily represent those of their affiliated organizations, or those of the publisher, the editors and the reviewers. Any product that may be evaluated in this article, or claim that may be made by its manufacturer, is not guaranteed or endorsed by the publisher.
